# A Lateral Flow-Recombinase Polymerase Amplification Method for *Colletotrichum gloeosporioides* Detection

**DOI:** 10.3390/jof10050315

**Published:** 2024-04-26

**Authors:** Wei-Teng Xu, Xin-Yu Lu, Yue Wang, Ming-Han Li, Ke Hu, Zi-Jie Shen, Xiao-Qin Sun, Yan-Mei Zhang

**Affiliations:** 1 Institute of Botany, Jiangsu Province and Chinese Academy of Sciences, Nanjing 210014, China; ecologyxwt@gmail.com (W.-T.X.); luxinyu@jib.ac.cn (X.-Y.L.); yuewang@cnbg.net (Y.W.); minghan_li@foxmail.com (M.-H.L.); 2022716005@stu.njau.edu.cn (K.H.); shenzijie@jib.ac.cn (Z.-J.S.); xiaoqinsun@cnbg.net (X.-Q.S.); 2 Jiangsu Key Laboratory for the Research and Utilization of Plant Resources, Nanjing 210014, China; 3 Jiangsu Provincial Science and Technology Resources Coordination Platform (Agricultural Germplasm Resources) Germplasm Resources Nursery of Medicinal Plants, Nanjing 210014, China

**Keywords:** *Dioscorea alata*, *Colletotrichum gloeosporioides*, primer development, pathogen identification, disease diagnosis

## Abstract

The greater yam (*Dioscorea alata*), a widely cultivated and nutritious food crop, suffers from widespread yield reduction due to anthracnose caused by *Colletotrichum gloeosporioides*. Latent infection often occurs before anthracnose phenotypes can be detected, making early prevention difficult and causing significant harm to agricultural production. Through comparative genomic analysis of 60 genomes of 38 species from the *Colletotrichum* genus, this study identified 17 orthologous gene groups (orthogroups) that were shared by all investigated *C. gloeosporioides* strains but absent from all other *Colletotrichum* species. Four of the 17 *C. gloeosporioides*-specific orthogroups were used as molecular markers for PCR primer designation and *C. gloeosporioides* detection. All of them can specifically detect *C. gloeosporioides* out of microbes within and beyond the *Colletotrichum* genus with different sensitivities. To establish a rapid, portable, and operable anthracnose diagnostic method suitable for field use, specific recombinase polymerase amplification (RPA) primer probe combinations were designed, and a lateral flow (LF)-RPA detection kit for *C. gloeosporioides* was developed, with the sensitivity reaching the picogram (pg) level. In conclusion, this study identified *C. gloeosporioides*-specific molecular markers and developed an efficient method for *C. gloeosporioides* detection, which can be applied to the prevention and control of yam anthracnose as well as anthracnose caused by *C. gloeosporioides* in other crops. The strategy adopted by this study also serves as a reference for the identification of molecular markers and diagnosis of other plant pathogens.

## 1. Introduction

*Dioscorea alata*, commonly known as greater yam, is a widely cultivated and highly important crop in tropical and subtropical areas. It is the most widely distributed and most-produced yam species globally, except for *D. polystachya* [1]. Greater yam is a widely cultivated tuber crop due to its high yield potential even in low soil fertility conditions, as well as its ease of propagation, early vigor in competing with weeds, being rich in starch and protein [2], and tuber storability [3]. However, the yields of greater yam have been hindered by various biotic and abiotic constraints. Among the diseases caused by microbial infection, anthracnose caused by *Colletotrichum gloeosporioides* is the main problem affecting greater yam yields and post-harvest quality [4]. Greater yam anthracnose can induce symptoms such as leaf necrosis, premature shedding, and wilting of young shoots in yam plants, resulting in yield reductions of up to 90% [5] and leading to significant economic losses, and this has necessitated frequent fungicide applications for chemical control. However, this approach poses environmental risks and potential fungicide resistance, and it is often cost-prohibitive for many farmers in tropical regions [6].

Moreover, recent studies have revealed high genotypic diversity within *C. gloeosporioides* [7]. It is a species complex of more than 20 subspecies with a rapid rate of intraspecific variation and a wide geographic distribution [8], suggesting potential for the evolution of new virulent strains capable of overcoming plant resistance. In addition, *C. gloeosporioides* is not only a widespread causal agent of greater yam anthracnose but also a pathogen infecting over 470 different host genera from monocotyledons to dicotyledons [9]. Controlling *C. gloeosporioides* disease has been hampered by its rapid spread and long-term survival in natural environments. The pathogen exhibits a relatively extended incubation period following its infection of the host plant, thereby impeding the activation of the plant’s immune responses [10]. Therefore, the rapid and accurate detection of *C. gloeosporioides* during the early infection stages is critical for disease management.

Traditionally, the identification and characterization of *Colletotrichum* spp. relied on morphological features such as the colony color, conidia and appressorium shape, and growth rate. Molecular techniques now provide alternative methods for taxonomic studies and are important tools for species delimitation [11,12]. The selection of appropriate target genes is essential for the successful detection of specific pathogens. The target genes should exhibit a high degree of conservation within the pathogen species to ensure broad applicability across different strains and isolates [13,14]. One established PCR-based method for detecting *C. gloeosporioides* is to detect the internally transcribed spacer (ITS) 1 region of ribosomal DNA (rDNA) [15]. However, this region has a low specificity for species discrimination within the *Colletotrichum* genera because of the high sequence identity of the ITS sequence among closely related species [16]. Therefore, mining new *C. gloeosporioides*-specific targets is needed for rapid and precise detection of this pathogen.

Comparative genomics is a powerful approach used to identify specific target genes for various applications, including diagnostics [17], classification [18], and evolutionary [19] studies. By comparing the genomes of different species or individuals within a species, researchers can identify genes that are unique to specific taxa [13,14], which may serve as ideal targets for designing species-specific diagnostic methods. Molecular techniques, including the polymerase chain reaction (PCR) and reverse transcription quantitative PCR (RT-qPCR), are highly sensitive DNA-based methods that have been applied in *C. gloeosporioides* detection [20,21]. However, conventional PCR-based methods are not suitable for field detection due to the need for bulky and expensive lab equipment and complicated protocols for amplification. Recombinase polymerase amplification (RPA) has been proven to be a promising alternative to traditional PCR-based methods for the detection of plant pathogens in recent studies [22]. Unlike the PCR, RPA does not require complicated temperature cycling conditions, making it more conducive for field applications. This isothermal amplification technique offers several advantages, including field mobility, rapid results, and the ability to perform amplification directly on a sample material without the need for DNA extraction [23]. Furthermore, the combination of RPA with lateral flow (LF) assays allows for rapid detection and easy visualization of results, enhancing its practicality for on-site testing [24]. The application of this technology also reduces the cost requirements for specialist and complicated instruments. Moreover, because the detection kit facilitates industrial production, its cost will be further reduced.

This study conducted comparative genomics to identify *C. gloeosporioides*-specific genes. The designed primer pairs targeting 4 out of the 17 *C. gloeosporioides*-specific genes display high specify and sensitivity in amplifying the genomic DNA of *C. gloeosporioides*. Using one gene as a target, we developed a rapid and simple LF-RPA method for the detection of *C. gloeosporioides*. The LF-RPA method enables detection of *C. gloeosporioides* in resource-limited laboratories and field environments at a low cost. Furthermore, the strategy adopted by this study may serve as a reference for the molecular mark development and identification of different kinds of plant pathogens.

## 2. Materials and Methods

### 2.1. Materials

In this study, 60 genomes from 38 species of the genus *Colletotrichum* were used in the comparative genomic analysis. The *C. gloeosporioides* strain CgDa01 (GCA_021650765.1), which was recently isolated from greater yam, was sequenced by our lab [25]. The other 59 *Colletotrichum* genomes were downloaded from the NCBI database (Table S1).

The fungal species included in this research for DNA extraction were *Fusarium graminearum*, *F. oxysporum*, *Sclerotinia sclerotiorum*, *Botrytis cinerea*, *Blumeria graminis*, *C. fructicola*, and *C. gloeosporioides* strains CgDa01 and CgDaM3. The CgDa01 and CgDaM3 strains were isolated in our laboratory. The *C. gloeosporioides* strains CgDa01 and CgDaM3 were kept in our lab. The *C. fructicola* strain was a gift from Dr. Yuqiang Zhao of the Fruit Tree Research Centre of the Institute of Botany at the Jiangsu Province and Chinese Academy of Sciences. The other strains were kindly provided by Professor Dao-Long Dou’s laboratory at Nanjing Agricultural University.

### 2.2. Comparative Genomic Analysis

The OrthoFinder software (version 2.5.2) was employed to analyze orthologous relationships across all coding genes of the genomes used in this study with the default settings [26]. Genes shared by all *C. gloeosporioides* strains but absent in the genomes of all other *Colletotrichum* species were categorized as *C. gloeosporioides*-specific orthogroups. The coding sequences (CDSs) of each orthogroup were aligned by using the ClustalW program (v1.4) integrated into BioEdit (v7.2.5) [27]. The resulting alignments were used for the next step: molecular marker design.

### 2.3. Fungal Cultivation and Genomic DNA Extraction 

All fungal strains were cultivated in a potato dextrose agar (PDA) medium and incubated at 28 °C for 5 days. Mycelium from the actively growing margin of the culture was carefully transferred to fresh plates to establish pure, isolated colonies through incubation under the same conditions. From these colonies, mycelium was introduced into a potato dextrose broth (PDB) medium and incubated for 2 days. Then, the culture was filtered, and the mycelium was collected, promptly frozen in liquid nitrogen, and ground to a fine powder. The genomic DNA was then extracted from the powdery mycelium using the modified hexadecyltrimethylammonium bromide (CTAB) method [28]. All reagents in this section were purchased from Solarbio Technology Co., Ltd. (Beijing, China). The DNA concentration was quantified using a spectrophotometer, and the DNA was stored at −20 °C for later use.

### 2.4. Primer Design and Specificity Identification

The primers (Table S2) were designed against the conserved region of the CDSs of the *C. gloeosporioides*-specific orthogroups based on the alignments [29]. To evaluate the specificity of the primers, PCR assays were performed. The total reaction volume for each PCR was 20 μL, including 10 μL of Taq Green Mix (sourced from Vazyme Biotechnology Co., Ltd., Nanjing, China), 0.5 μL of each forward and reverse primer (concentration of 10 μmol/L), 1 μL of the template DNA (concentration at 100 ng/μL), and 8 μL of deionized distilled water (ddH_2_O). The PCR conditions included an initial denaturation step at 95 °C for 5 min followed by 35 cycles, with each cycle consisting of denaturation at 95 °C for 15 s, annealing at 58 °C for 15 s, and extension at 72 °C for 30 s. A final extension was carried out at 72 °C for 10 min. The PCR products were then subjected to analysis via 1% agarose gel electrophoresis. The primers that demonstrated a specific affinity for targeting *C. gloeosporioides* were selected for further study.

### 2.5. Sensitivity Testing of Primers

For the sensitivity assessment, DNA extracted from the *C. gloeosporioides* strain CgDa01 was used as the template. A series of serial dilutions was performed to obtain DNA concentrations of 10 ng/μL, 1 ng/μL, 0.1 ng/μL, and down to 10^−5^ ng/μL. The conditions for the amplification and gel analysis were kept consistent as described above.

### 2.6. Preparing DNA Extraction of Yam Anthracnose Samples

The plant inoculation protocol [30] involved harvesting conidia from 7 day-old CgDa01 cultures. For this, 3 mL of sterile water was added to a 60 mm Petri dish, and the mycelium surface was gently scraped until it was detached with the aid of a spreader. The resulting slurry was filtered through four layers of gauze into a centrifuge tube, repeating the process as many times as necessary to ensure comprehensive capture of the conidia. The conidial concentration was quantified using a hemocytometer and adjusted to 10^5^ conidia/mL. Tween-20 (Solarbio Technology Co., Ltd., Beijing, China), at a final concentration of 0.1%, was added to the suspension as an emulsifying agent. Subsequently, the leaves of greater yam were disinfected with alcohol, punctured with a syringe, and inoculated with 10 µL of the conidial emulsion at the puncture site. These leaves were then maintained in a humidified environment inside plastic containers padded with moist cotton and incubated at 25 °C for 5 days. Tissue samples from the inoculated area were collected on days 0 (immediately after inoculation), 1, 3, and 5 post inoculation. After washing with ddH_2_O to remove the conidia at the surface, the leaf samples were subjected to DNA extraction and subsequent PCR analysis. For comparison, the control group was inoculated with sterile water alone.

### 2.7. Development of a Rapid RPA Detection Kit against C. gloeosporioides

For RPA detection, the TwistAmp^®^ DNA amplification kit (TwistDx, Maidenhead, UK) was employed. The primers and probes were designed while following the recommendations of the kit’s manual. The template DNA was serially diluted to a final concentration of 1 fg/μL. The RPA reaction was configured in a 50 μL mixture containing 2.1 μL of both forward and reverse primers at a concentration of 10 μmol/L, 0.6 μL of the Twist Amp LF probe at a 10 μmol/L concentration, 29.5 μL of the provided buffer, 5 μL of the target DNA, 8.2 μL of ddH_2_O, and finally 2.5 μL of magnesium acetate at a concentration of 280 mmol/L. After thoroughly mixing the components, the mixture was incubated at 37 °C for 4 min. Following another round of mixing, the sample was incubated again at 37 °C for an additional 20 min. Detection was then executed using a lateral flow strip. Specifically, 0.5 mL of the reaction mixture was applied to the sample well of the lateral flow strip and allowed to react at ambient temperature for 10 min before assessing the results. Similarly, we applied this protocol to the DNA samples obtained in Section 2.6, which were extracted from leaves of greater yam inoculated by *C. gloeosporioides* at different time points.

## 3. Results

### 3.1. Comparative Genomic Analsysis of 60 Genomes Revealed C. gloeosporioides-Specific Orthogroups

**Figure 1:**
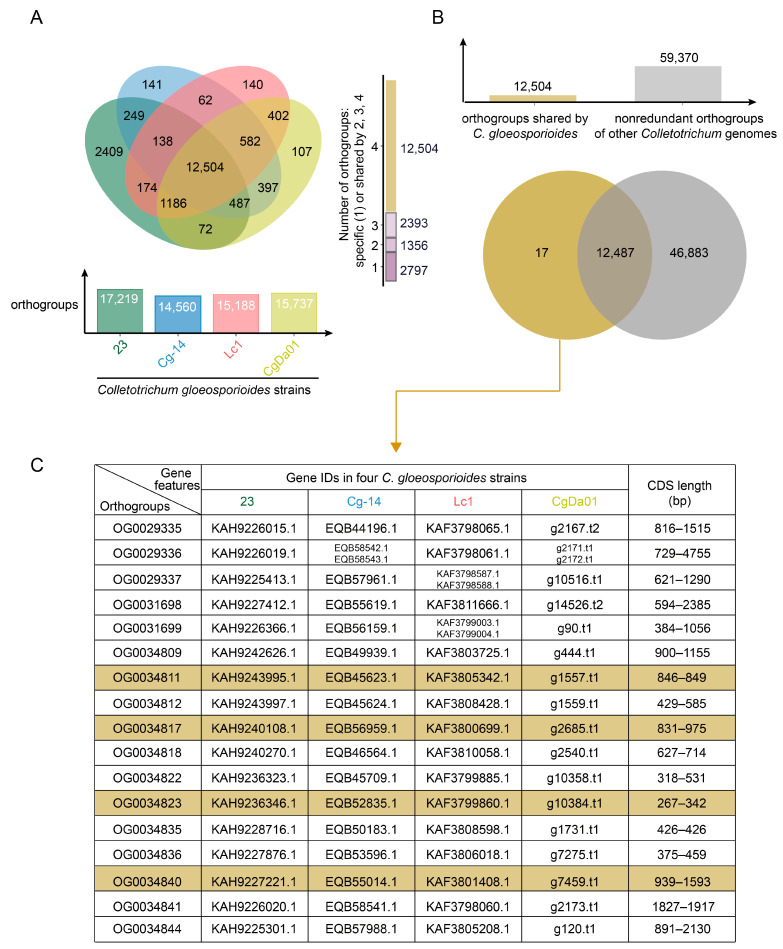
Statistics of orthologous gene clustering analyses. (**A**) Number of orthogroups occupied by genes of different *C. gloeosporioides* strains, Venn diagram shows the number of orthogroups which were strain-specific (1) or shared by 2, 3, or 4 strains. (**B**) Comparison of orthogroups shared by all *C. gloeosporioides* strains with all non-redundant orthogroups of the other 56 *Colletotrichum* genomes. (**C**) Details of *C. gloeosporioides*-specific orthogroups, including gene IDs and length of each genome. The four colored orthogroups were eventually the molecular markers used in this study.

To identify genes that were shared by all *C. gloeosporioides* strains but absent in all other *Colletotrichum* species, comparative genomic analysis was conducted on 4 genomes of *C. gloeosporioides* and 56 genomes from 37 other species of the *Colletotrichum* genus. The results showed that the genes from the four *C. gloeosporioides* strains could be assigned to 19,050 orthogroups (Figure 1A and Table S3). Among them, 12,504 orthogroups were shared by all of the four investigated *C. gloeosporioides* genomes, whereas 2393 and 1356 orthogroups were shared by three and two genomes, respectively, and 2797 orthogroups were strain-specific (Figure 1A). Further comparative analysis of the 12,504 *C. gloeosporioides*-conserved orthogroups with the 59,370 orthogroups identified from other *Colletotrichum* genomes revealed that 12,487 orthogroups were present in both *C. gloeosporioides* and other *Colletotrichum* species, while 17 orthogroups were *C. gloeosporioides*-specific (Figure 1B and Table S3). A detailed analysis of the genes belonging to the 17 orthogroups revealed that 14 of them were presented as single-copy genes in all *C. gloeosporioides* strains (Figure 1C), suggesting their potential application in molecular detection of *C. gloeosporioides*. 

### 3.2. Screening of Specific Primers for C. gloeosporioides Detection

By aligning the CDSs of the genes belong to the 14 *C. gloeosporioides*-specific orthogroups, highly conserved regions that are suitable for PCR primer design were identified from the four orthogroups, namely OG0034811, OG0034817, OG0034823, and OG0034840. The protein encoded by genes belonging to the orthogroup OG0034817 was annotated as Ribonuclease H, whereas proteins encoded by genes from the remaining orthogroups were all annotated as uncharacterized proteins. The specific primers were then designed against genes belonging to these orthogroups by targeting the conserved regions. Within these orthogroups, the lengths of the target sequences were 542 bp (OG0034840), 156 bp (OG0034823), 691 bp (OG0034817), and 327 bp (OG0034811).

**Figure 2:**
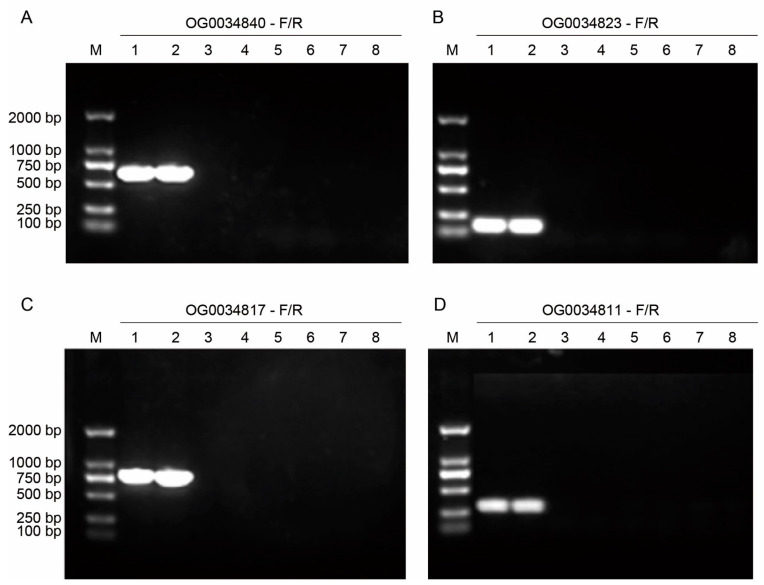
Determination of the primers targeting four *C. gloeosporioides*-specific orthogroups by PCR. M = 2000 bp DNA Ladder; 1 = *C. gloeosporioides* strain CgDa01; 2 = *C. gloeosporioides* strain CgDaM3; 3 = *Fusarium graminearum*; 4 = *F. oxysporum*; 5 = *Sclerotinia sclerotiorum*; 6 = *Botrytis cinerea*; 7 = *Blumeria graminis*; and 8 = *C. fructicola*. (**A**–**D**) PCR results of different primer pairs.

Initially, we performed a PCR assay using genomic DNA from eight strains as templates, including two *C. gloeosporioides* strains, one *C. fructicola* strain from the *Colletotrichum* genus, and five strains from fungal species outside of the *Colletotrichum* genus. The target products of the primer pairs targeting all of the four orthogroups successfully amplified the products from the genomic DNA of two *C. gloeosporioides* strains, with one specific band corresponding to each gene being detected in the agarose gel electrophoresis. In contrast, no bands were visualized in the PCR products of other species within and beyond the *Colletotrichum* genus. The results were consistent among the three replications, suggesting that the primer pairs targeting the four *C. gloeosporioides*-specific orthogroups OG0034840 (Figure 2A), OG0034823 (Figure 2B), OG0034817 (Figure 2C), and OG0034811 (Figure 2D) can specifically amplify DNA fragments from *C. gloeosporioides*.

### 3.3. Sensitivity Testing of Primers for C. gloeosporioides Detection

**Figure 3:**
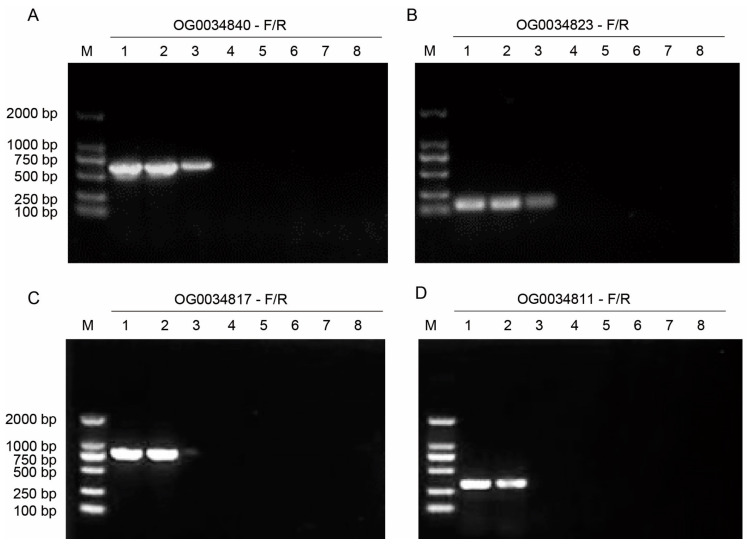
Sensitivity testing of four pairs of specific primers. M = 2000 bp DNA Ladder, and 1~8 = different DNA concentrations: 1 ng/μL, 100 pg/μL, 10 pg/μL, 1 pg/μL, 100 fg/μL, 10 fg/μL, 1 fg/μL, and NTC (no-template control), respectively. (**A**–**D**) PCR results of different primer pairs.

To evaluate the sensitivity of the primers targeting the OG0034840, OG0034823, OG0034817, and OG0034811 orthogroup genes, PCR analyses were performed using *C. gloeosporioides* strain CgDa01 genomic DNA as templates by diluting them into different concentrations from 1 ng/μL to 1 fg/μL. The results showed that the primer pairs of OG0034840 and OG0034823 could successfully amplify the target fragments at a genomic DNA concentration of 10 pg/μL (Figure 3A,B), whereas the primer pairs of OG0034817 and OG0034811 could only amplify the target fragments at a genomic DNA concentration of 100 pg/μL (Figure 3C,D). This experiment was replicated three times with similar results, which indicated that the primer pairs of OG0034840 and OG0034823 were more sensitive than those of OG0034817 and OG0034811 and might be more suitable for the development of rapid detection methods.

### 3.4. Evaluation of Specific Primers for C. gloeosporioides Detection in Infected Plant Tissues

**Figure 4:**
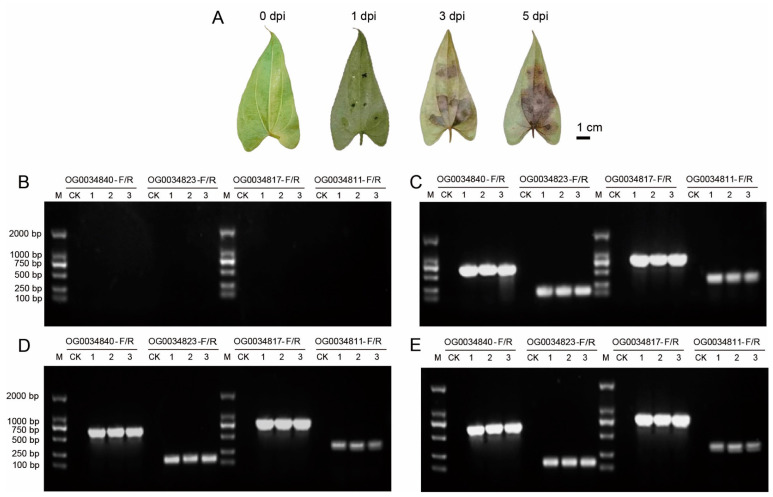
Evaluation of specific primers for *C. gloeosporioides* detection of infected plant tissues. (**A**) Phenotypes of leaves post inoculation with *C. gloeosporioide* on different days. Dpi = days post infection. (**B**–**E**) PCR results of DNA extracted from plant leaves at 0, 1, 3, and 5 days post inoculation (dpi) with *C. gloeosporioides*, respectively. M = 2000 bp DNA Ladder; CK = control check, namely greater yam leaves that were not inoculated with *C. gloeosporioides*; Lanes 1~3 = independent samples infected by *C. gloeosporioide* at the same time point.

To evaluate the efficiency of the four pairs of specific primers in the anthracnose yam samples, greater yam leaves exhibiting different anthracnose phenotypes were collected at one, three, and five days post inoculation with the *C. gloeosporioides* strain CgDa01 (Figure 4A). The greater yam leaves that were not inoculated with *C. gloeosporioides* were used as controls. DNA from the CgDa01-inoculated or non-inoculated leaves were extracted and used as templates for the PCR analysis. The results show that all of the four primer pairs failed to amplify any products when using the DNA of the yam leaves not inoculated with CgDa01 or the DNA of yam leaves inoculated with CgDa01 at 0 dpi as templates (Figure 4B). In contrast, all of the four primer pairs successfully amplified the target fragments from the DNA samples of yam leaves infected with *C. gloeosporioides* at 1, 3, and 5 dpi (Figure 4C–E). The results indicate that these specific primer pairs can detect the infection of yams by *C. gloeosporioides* as early as the first day post infection.

### 3.5. Development of the LF-RPA Method for Rapid Detection of C. gloeosporioides

**Figure 5:**
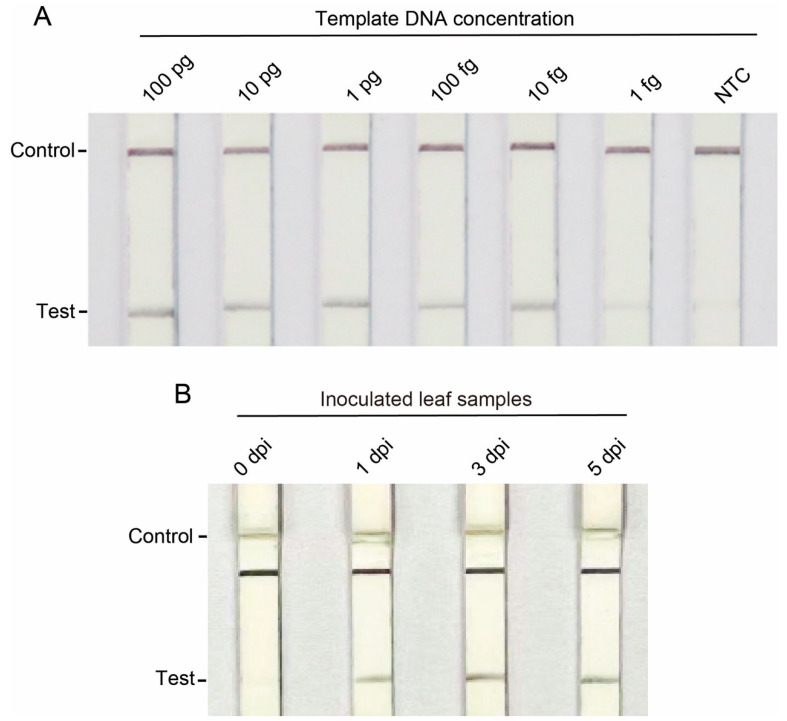
Sensitivity test of LF-RPA *C. gloeosporioides* detection methods. NTC = no template control; Dpi = days post infection. (**A**) Sensitivity of LF-RPA at different concentrations of *C. gloeosporioides* genomic DNA. (**B**) Results of LF-RPA conducted on DNA samples extracted from greater yam leaves infected by *C. gloeosporioides* from 0 to 5 dpi.

Having observed that all of the four primer pairs could specifically detect *C. gloeosporioides*, and the primer pairs of OG0034840 and OG0034823 were more sensitive than those of OG0034817 and OG0034811, we selected OG0034840 as a target for developing an LF-RPA based *C. gloeosporioides* detection method. The detection threshold of the LF-RPA assay was measured using serial dilutions of genomic DNA of *C. gloeosporioides* as templates. The results reveal that a clear band could be detected at a template concentration of 10 fg/μL and a faint band was present for the 1 fg/μL template, with all quality control lines being clearly discernible (Figure 5A). Therefore, the detection threshold of the LF-RPA assay was determined to be down toward 10 fg and even 1 fg of genomic DNA, which is more sensitive than conventional PCR in the detection of *C. gloeosporioides* genomic DNA. Next, we used a lateral flow strip to detect the DNA obtained from the greater yam leaves infected or not infected by *C. gloeosporioides* as described in Section 3.4. Test bands were clearly observed in the DNA extracted from the greater yam leaves inoculated with *C. gloeosporioides* at 1, 3, and 5 dpi but not in the DNA of the greater yam leaves that were inoculated with *C. gloeosporioides* at 0 dpi. The experiments were repeated three times with consistent results, suggesting that the LF-RPA assay can detect *C. gloeosporioides* in infected leaves after the first day of inoculation (Figure 5B).

## 4. Discussion and Conclusions

*C. gloeosporioides* is a causal agent of anthracnose, a plant disease that is economically important and widespread [31]. Developing a rapid and user-friendly anthracnose diagnosis method is highly important and valuable for the cultivation of greater yam and other cash crops. In this study, we performed a genome-wide screening of *C. gloeosporioides*-specific sequences and designed amplification primers, which were then validated for specificity, sensitivity, and *C. gloeosporioides*-infected plant sample detection. Finally, we developed a practical LF-RPA detection kit.

Species-specific genes are ideal targets for developing molecular detection kits against microbes [13,14]. However, rapid evolution of a microbe genome often causes the presence or absence of variation in genes among different strains of the same species [32]. To overcome this problem, comparative genomic analysis has been adopted by independent studies to identify species-specific genes. For example, a recent study identified species-specific genes, namely *ComFA*, *group_14348*, *group_26190*, and *group_26478* from *Staphylococcus aureus*, *S. epidermidis*, *S. haemolyticus*, and *S. hominis*, respectively, as molecular markers and used these markers for developing molecular detection methods against these pathogens [33]. Similarly, a series of species-specific genes was obtained for seven species belonging to the *Cronobacter* genus through pan-genome analysis, including 4, 10, 31, 30, 70, 93, and 331 genes specific to *Cronobacter sakazakii*, *Cr. malonaticus*, *Cr. dublinensis*, *Cr. turicensis*, *Cr. muytjensii*, *Cr. universalis*, and *Cr. condimenti*, respectively [34]. Inspired by these previous reports, this study included genomes of four *C. gloeosporioides* strains that were isolated from different hosts by independent studies (Table S1). The identification of orthogroups shared by all investigated strains suggests their conservation during the evolution of *C. gloeosporioides*. Considering *C. gloeosporioides* has dozens of closely related species within the same genus [35], we included the genomes of 37 other species belonging to the *Colletotrichum* genus. This step enabled us to identify 17 orthogroups that are specific to and conservatively shared by all *C. gloeosporioides* strains. These orthogroups provide a raw resource for the screening of gene targets for developing molecular detection methods. Although these 17 orthogroups display the same feature of being shared by all *C. gloeosporioides* strains, they may exhibit different evolutionary patterns and show differential sequence variation. Therefore, a further sequence alignment assay enabled us to identify conserved sequence regions from the four orthogroup genes.

Our results show that while the primer pairs of all of the above four orthogroups could specifically detect *C. gloeosporioides* out of the pathogens within the *Colletotrichum* genus, the primer pairs of OG0034840 and OG0034823 exhibited greater sensitivity with a detection threshold of 10 pg/μL. These primers produced clear, discrete bands at this concentration. In contrast, the other two primer pairs targeting OG0034817 and OG0034811 could only detect the template DNA at a concentration of 100 pg/μL, which may have been due to their higher annealing temperature and easier-to-form secondary structures. These two primer pairs can be used as an alternative to those targeting OG0034840 and OG0034823 for the specific detection of *C. gloeosporioides*. By using the above primer pairs to detect *C. gloeosporioides* in yam leaves, we found that all of them could detect infections of the *C. gloeosporioides* strain CgDa01 as early as the first day post incubation when the leaves did not yet have any obvious external symptoms. Therefore, our approach can serve as an effective method for the early diagnosis of yam anthracnose, which is conducive to reducing the spread of the disease and improving the yield and quality of greater yam.

Currently, methods for identifying *C. gloeosporioides* and its close relatives can be technically divided into two main categories: microscopic detection techniques based on the morphology of the organism and detection techniques based on PCR amplification of nucleic acid sequences [15]. Because morphological identification is easily influenced by environmental factors, the latter is used more nowadays. In the host greater yam, Mithun Raj et al. previously used nested PCR to detect a *C. gloeosporioides* limit of 200 fg/μL [36], and a recent study showed that RT-qPCR in other hosts such as *Arabidopsis thaliana* could increase the detection limit to 5 fg/μL [37]. Compared with the above PCR techniques, the LF-RPA detection method developed in this study can recognize *C. gloeosporioides* DNA at even a mere 1 fg/μL. Moreover, a recent study proposed a DNA extraction protocol that does not require complicated instruments and does not rely on the participation of specialists. The protocol can be completed in only 30 s and costs only USD 0.06 per session [38]. This method can be jointly applied to our study to improve the efficiency of extracting DNA in a resource-limited environment. In addition, the LF-RPA detection kit also exhibits the advantage of a low cost, being as low as USD 0.61 per reaction [39]. Because the industrial production of lateral flow strips, the key component, is well established, they can easily be applied in the field and poor rural areas.

In summary, through the utilization of comprehensive comparative genomic analysis, we successfully obtained distinct molecular markers specific to *C. gloeosporioides* and conservatively shared by different strains. In taking advantage of these molecular markers, we developed an efficient, rapid, transportable, and simple method by introducing RPA technology to detect *C. gloeosporioides* in greater yam for the first time. This enables early diagnosis of yam anthracnose and establishes the groundwork for the prevention and control of this disease. The strategy adopted by this study also provides new research ideas for the development of detection and diagnosis tools for different fungal diseases.

## Data Availability

Data are contained within the article and Supplementary Materials.

## Supplementary Materials

The following supporting information can be downloaded at https://www.mdpi.com/article/10.3390/jof10050315/s1. Table S1: Genomes used in this study. Table S2: Candidate gene primer sequences. Table S3: Orthogroups identified from 60 *Colletotrichum* genomes.
