# Supplementary material for: A Lateral Flow-Recombinase Polymerase Amplification Method for Colletotrichum gloeosporioides Detection
Source: J Fungi (Basel). 2024 Apr 26;10(5):315. doi: 10.3390/jof10050315 (PMC11121841; doi:10.3390/jof10050315)
Supplement: Supplementary file 1 [file jof-10-00315-s001.zip › Table S1 Genomes used in this study.pdf]

Table S1. Genomes used in this study.

No.	Specie	NCBI ID	Strain	Size (Mbp)
1	*Colletotrichum gloeosporioides*	GCA_021432615.1	23	58.84
2	*Colletotrichum gloeosporioides*	GCA_000446055.1	Cg-14	53.21
3	*Colletotrichum gloeosporioides*	GCA_011800055.1	Lc1	61.90
4	*Colletotrichum gloeosporioides*	GCA_021650765.1	CgDa01	62.78
5	*Colletotrichum chlorophyti*	GCA_001937105.1	NTL11	52.39
6	*Colletotrichum filicis*	GCA_023376865.1	CBS 101611	62.97
7	*Colletotrichum fioriniae*	GCA_000582985.1	PJ7	49.00
8	*Colletotrichum fructicola*	GCA_009771025.1	CGMCC3.17371	58.06
9	*Colletotrichum fructicola*	GCA_012932255.1	Cg38 S1	58.95
10	*Colletotrichum fructicola*	GCA_013201875.1	CfS4	57.43
11	*Colletotrichum fructicola*	GCA_013201905.1	Cf415	56.01
12	*Colletotrichum fructicola*	GCA_013201925.1	Cf245	56.06
13	*Colletotrichum fructicola*	GCA_013390205.1	Cf413	56.53
14	*Colletotrichum fructicola*	GCA_000319635.1	Nara gc5	55.61
15	*Colletotrichum fructicola*	GCA_000319635.2	Nara gc5	59.54
16	*Colletotrichum abscissum*	GCA_023376855.1	Ca142	54.00
17	*Colletotrichum aenigma*	GCA_013390185.1	Cg56	59.19
18	*Colletotrichum asianum*	GCA_009806415.1	ICMP 18580	64.73
19	*Colletotrichum camelliae*	GCA_011947485.2	CcLH18	57.80
20	*Colletotrichum graminicola*	GCA_000149035.1	M1.001	51.60
21	*Colletotrichum higginsianum*	GCA_000313795.2	IMI 349063	49.08
22	*Colletotrichum higginsianum*	GCA_004920355.1	MAFF30563	49.79
23	*Colletotrichum higginsianum*	GCA_023705605.1	MAFF 245053	49.07
24	*Colletotrichum higginsianum*	GCA_001672515.1	IMI 349063	50.72
25	*Colletotrichum incanum*	GCA_001625285.1	MAFF 238704	53.60
26	*Colletotrichum incanum*	GCA_001855235.1	MAFF238712	53.25
27	*Colletotrichum karsti*	GCA_011947395.2	CkLH20	51.85
28	*Colletotrichum liriopes*	GCA_022179045.1	MAFF 242679	52.97
29	*Colletotrichum lupini*	GCA_023278565.1	IMI 504893	63.41
30	*Colletotrichum musicola*	GCA_014235935.1	LFN0074	52.73
31	*Colletotrichum nymphaeae*	GCA_001563115.1	SA-01	49.96
32	*Colletotrichum orbiculare*	GCA_000350065.2	104-T	89.75
33	*Colletotrichum orchidophilum*	GCA_001831195.1	IMI 309357	48.56
34	*Colletotrichum plurivorum*	GCA_014235945.1	LFN00145	49.70
35	*Colletotrichum salicis*	GCA_001563125.1	CBS 607.94	48.37
36	*Colletotrichum scovillei*	GCA_011075155.1	TJNH1	52.00
37	*Colletotrichum scovillei*	GCA_018906675.1	Coll-365	49.92
38	*Colletotrichum scovillei*	GCA_018906765.1	Coll-153	50.11
39	*Colletotrichum scovillei*	GCA_018907675.1	Coll-524	51.49
40	*Colletotrichum shisoi*	GCA_006783085.1	PG-2018a	69.67
41	*Colletotrichum siamense*	GCA_013201745.1	CAD2	58.15
42	*Colletotrichum siamense*	GCA_013201755.1	CAD5	58.40

43	*Colletotrichum siamense*	GCA_013201795.1	CAD4	58.15
44	*Colletotrichum siamense*	GCA_013201865.1	CAD1	58.40
45	*Colletotrichum siamense*	GCA_013390195.1	Cg363	62.94
46	*Colletotrichum sidae*	GCA_004367935.1	CBS 518.97	86.83
47	*Colletotrichum simmondsii*	GCA_001563135.1	CBS122122	50.47
48	*Colletotrichum sojae*	GCA_014235955.1	LFN0009	49.35
49	*Colletotrichum spaethianum*	GCA_022836535.1	MAFF 239500	50.92
50	*Colletotrichum spinosum*	GCA_004366825.1	CBS 515.97	82.73
51	*Colletotrichum sublineola*	GCA_000696135.1	TX430BB	46.76
52	*Colletotrichum tanaceti*	GCA_005350895.1	BRIP57314	57.91
53	*Colletotrichum tofieldiae*	GCA_001625265.1	0861	52.84
54	*Colletotrichum tofieldiae*	GCA_022836555.1	MAFF 712333	54.25
55	*Colletotrichum tofieldiae*	GCA_022836575.1	MAFF 712334	53.98
56	*Colletotrichum tofieldiae*	GCA_022836595.1	0861	52.99
57	*Colletotrichum trifolii*	GCA_004367215.1	543-2	109.7
58	*Colletotrichum tropicale*	GCA_013201785.1	CgS9275	55.85
59	*Colletotrichum truncatum*	GCA_014235925.1	CMES1059	56.10
60	*Colletotrichum viniferum*	GCA_013201765.1	CGW01	68.45
