# Supplementary material for: A Lateral Flow-Recombinase Polymerase Amplification Method for Colletotrichum gloeosporioides Detection
Source: J Fungi (Basel). 2024 Apr 26;10(5):315. doi: 10.3390/jof10050315 (PMC11121841; doi:10.3390/jof10050315)
Supplement: Supplementary file 1 [file jof-10-00315-s001.zip › Table S2 Primer sequences.pdf]

Table S2 Candidate gene primer sequences

Primer name	Primer sequence (5'-3')
Cg-OG0034811-F	CTGAACTTGCACTGCAGCAA
Cg-OG0034811-R	CCACACGATGCACCTCTGTT
Cg-OG0034817-F	CGTTCGTTGCAGAGCCTAAC
Cg-OG0034817-R	GCAGCATTCACCGCAAGTTT
Cg-OG0034823-F	TGGAAACGGGAACTTGGTCAG
Cg-OG0034823-R	CCGTCGTGCAAACCAACAG
Cg-OG0034840-F	AGGCAGCTTAGCAATCCGAA
Cg-OG0034840-R	CGGAGAATCTCTGCAAGGGG
